# The hygiene hypothesis for allergy – conception and evolution

**DOI:** 10.3389/falgy.2022.1051368

**Published:** 2022-11-24

**Authors:** Michael R Perkin, David P Strachan

**Affiliations:** Population Health Research Institute, St. George's, University of London, London, United Kingdom

**Keywords:** hygiene hypothesis, allergy epidemic, birth order, hay fever (H.F), hygiene, microbiome

## Abstract

In 1989, a short paper entitled “Hay fever, hygiene and household size” observed that British children from larger families were less likely to develop hay fever and suggested that this could be because early exposure to infection prevents allergy. This sibship size association for hay fever, since replicated many times in Britain and other affluent countries and confirmed by objective measures of atopy, prompted what has come to be known as the “hygiene hypothesis for allergy”, although that term was not specifically used in the 1989 paper. The present paper reviews the historical roots of the “hygiene hypothesis” and charts its development over more than 30 years. Initial scepticism among immunologists turned to enthusiasm in the mid-1990s as the Th1/Th2 paradigm for allergic sensitisation emerged from animal experiments and the concept of “immunological old friends” became popular from the early 2000s. From the late 1990s, observations of reduced allergy risk among children of anthroposophic families and those brought up on farms suggested that the sibship size effects formed part of a broader range of “hygiene-related” determinants of allergy. Children from large families with farming exposure have approximately sixfold reduction in prevalence of hay fever, indicating the potential strength and epidemiological importance of these environmental determinants. During the 21st century, a wide range of specific microbial, environmental and lifestyle factors have been investigated as possible underlying mechanisms, but sadly none have emerged as robust explanations for the family size and farming effects. Thus, while the “hygiene hypothesis” led to a fundamental reappraisal of our relationship with our microbial environment and to the concept that early exposure, rather than avoidance, is beneficial for developing a healthy immune system, the underlying mechanism for variations in allergy prevalence with family size remains, in Churchillian terms, “a riddle wrapped in a mystery inside an enigma”.

## The “hygiene hypothesis”

**Figure 1:**
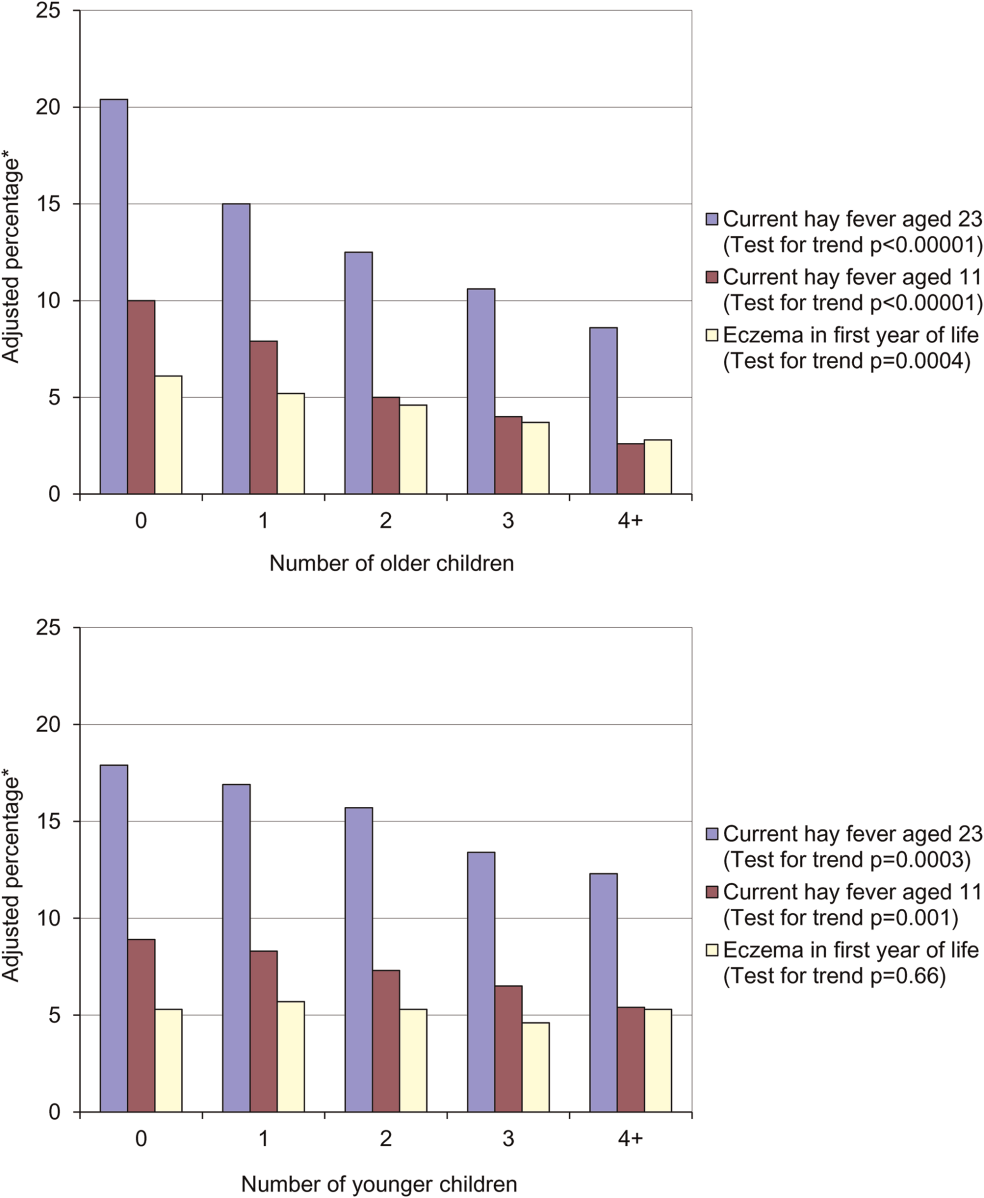
Prevalence of hay fever and eczema by number of older children (top panel) and number of younger children (bottom panel) in the household at age 11. *Adjusted for number of younger children in household, father's social class, housing tenure and shared household amenities in childhood, breastfeeding, region of birth and cigarette smoking at 23.

In 1989 Strachan published a paper entitled “Hay fever, hygiene and household size”, in which he observed that increasing family size was associated with a reduced risk of developing hay fever (1). Using data from a British 1958 birth cohort (the 1958 National Child Development Study) he found that the prevalence of hay fever at both 11 (parent-reported) and 23 (self-reported) years of age significantly decreased with increasing number of older children living in the same household (Figure 1, top panel).

The association between parent recalled eczema in the first year of life and the number of older children was also statistically significant. Strachan hypothesised that allergic diseases were being prevented by infection in early childhood – the “hygiene hypothesis”. He postulated that these infections were being acquired through:

• Transmission by unhygienic contact with older siblings, or
• Prenatal acquisition from a mother infected by contact with her older children.

A similar, but weaker, trend was seen with the number of younger children in the household that was statistically significant for hay fever at both 11 and 23 years of age (Figure 1, bottom panel). As expected, there was no association between the number of younger siblings in the household and parent reported eczema in the first year of life. On the basis of the association between presence of younger siblings and hay fever he proposed that:

• Later infection or reinfection by younger siblings might confer additional protection against hay fever.

He suggested that the emergence of allergic disease in the twentieth century as a “post-industrial revolution epidemic” (2) could be related to the combined effect of a number of factors: declining family size, improvements in household amenities, and higher standards of personal cleanliness. Together these may have:

“….reduced the opportunity for cross infection in young families. This may have resulted in more widespread clinical expression of atopic disease, emerging earlier in wealthier people, as seems to have occurred for hay fever.”

## Hygiene hypothesis – historical precedent

This paucity of hay fever amongst the poor had been observed by Bostock. Bostock first described hay fever or “summer catarrh” on the basis of his own symptoms in 1819. It took another 9 years for him to acquire 28 cases of the condition. In the publication describing these cases he observed:

“Summer catarrh… only occurs in the middle or upper classes of society, some indeed of high rank. I have made inquiry at the various dispensaries in London and elsewhere, and I have not heard of a single unequivocal case occurring amongst the poor.” (3)

**Figure 2:**
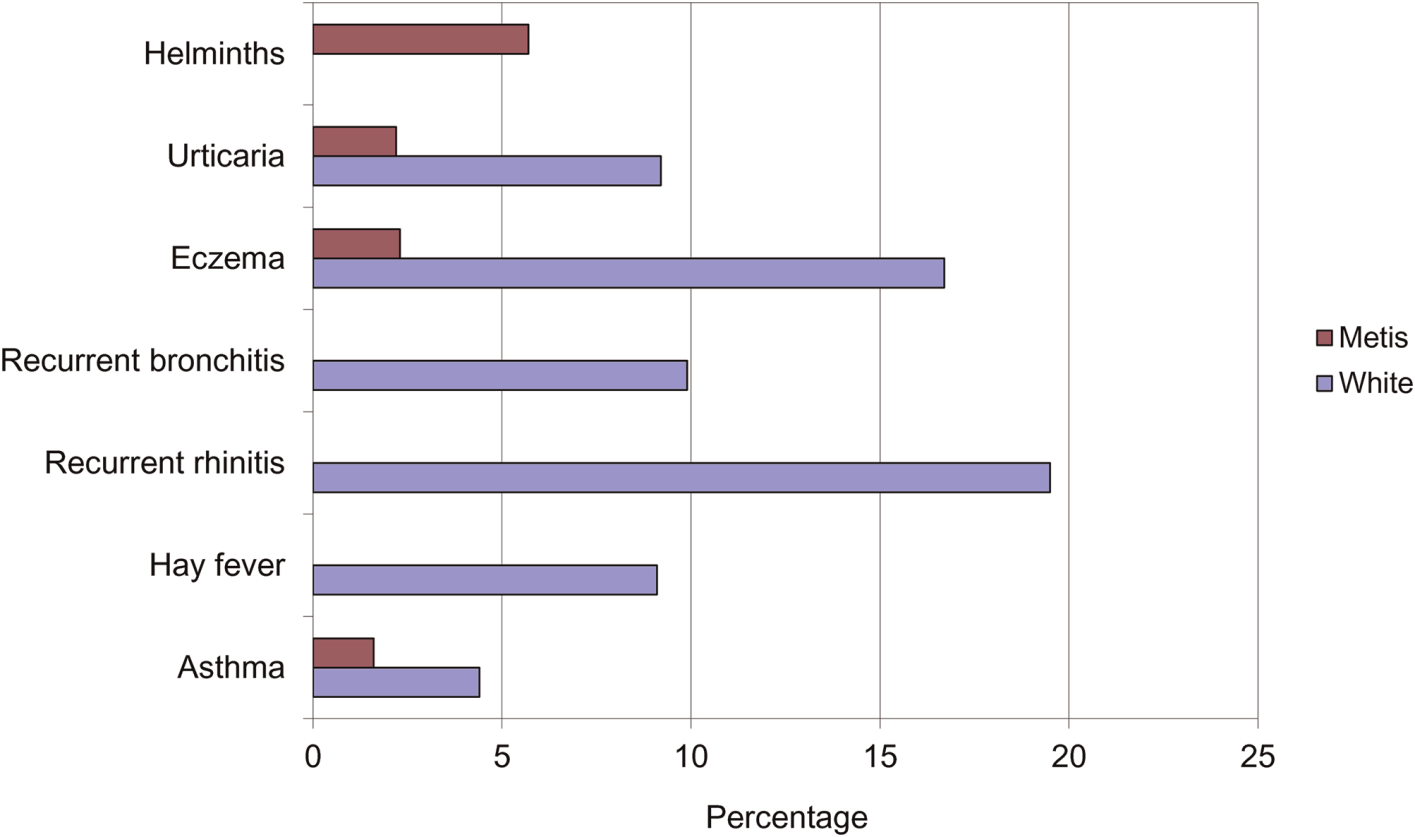
Prevalence of helminths infection and allergic disease in the Metis and white populations of Saskatchewan.

However, it was a paper by Gerrard in 1976 that suggested the inverse association between infections and atopy (4). He compared the prevalence of allergic disease in a native American population living in impoverished conditions, the Metis, with their neighbouring white community. Gerrard observed that despite the impoverishment of the Metis, exemplified in their helminth carriage, they had significantly less urticaria, eczema and asthma than the white population (Figure 2). Intriguingly, their levels of bronchitis and rhinitis were unrecordable because they were so susceptible to upper respiratory tract infections with coryza and coughs.

Total immunoglobulin E (IgE) levels were also noted to be significantly higher in the Metis community), independent of current helminth infection. From this data he reached the prescient conclusion:

“It is suggested that atopic disease is the price paid by some members of the white community for their relative freedom from diseases due to viruses, bacteria and helminths.”

The term “hygiene hypothesis” was first coined by Barker. In a 1985 paper analysing time trends of appendicitis he reached a similar conclusion to Gerrard, observing that:

“*The appendicitis epidemic in Britain started at the time when infections had begun to decline steeply, in consequence of improved living standards and sanitation. It may be postulated that* decreased *incidence of infection among children, especially in wealthier families, changed their pattern of immunity so that they responded to certain enteric or respiratory infections with lymphoid hyperplasia-which included the lymphoid tissue in the wall of the appendix*.”(5)

He concluded that his hypothesis explained the international distribution of appendicitis:

“Countries where it is common are characterised by lower incidences of infectious disease. The rising incidence of appendicitis among the more affluent, urbanised communities within non-industrialised countries may be explained by the improvement in the hygienic conditions of these communities.” (5)

Two years later, the term “hygiene hypothesis” appeared in the title or abstract of a research article for the first time (6). Exploring rates of appendicitis over time in the Eire and England, Scotland and Wales, the authors observed a positive relationship between markers of infection (mortality from enteric and respiratory infections, general practice consultations for infective disease and postneonatal mortality) and rates of appendicitis. The introduction stated:

“*Interest in the hypothesis that appendicitis is primarily an infective disorder has therefore revived. In particular, it has been suggested that the rise in appendicitis was a consequence of the Public Health Acts at the end of the last century. The resulting improvements in hygiene greatly reduced young children's exposure to enteric organisms and, it is argued, could thereby have altered their responses to infection in later years in such a way that acute appendicitis was triggered*.” (6)

In 1988, a year before the Strachan paper, Barker explored his “hygiene hypothesis” further (7). In three national samples of British children the relationship between appendicectomy and household amenities, crowding in the house and social class was investigated. He found that the risk of having an appendicectomy was related to amenities in the home and a household without a bathroom was associated with a reduced relative risk of appendicitis. Interestingly, for the 1970 cohort there was a positive association between increasing numbers of children aged under 16 in the household and risk of appendicectomy: 1 child in household RR 0.6, 2 children 0.7, 3 children 1.2, ≥4 children 1.9 (each additional child relative risk 1.6 (95% CI 1.2–2.0) (7). This wasn't explored further beyond considering increased sibship size a proxy for household overcrowding. He concluded that:

“*The findings support a relation between acute appendicitis and Western hygiene, which would explain the geographical distribution of the disease and its changing incidence over time. In the developing world, where children grow up in conditions of poor hygiene, there may be outbreaks of appendicitis when housing improves*.” (7)

**Figure 3:**
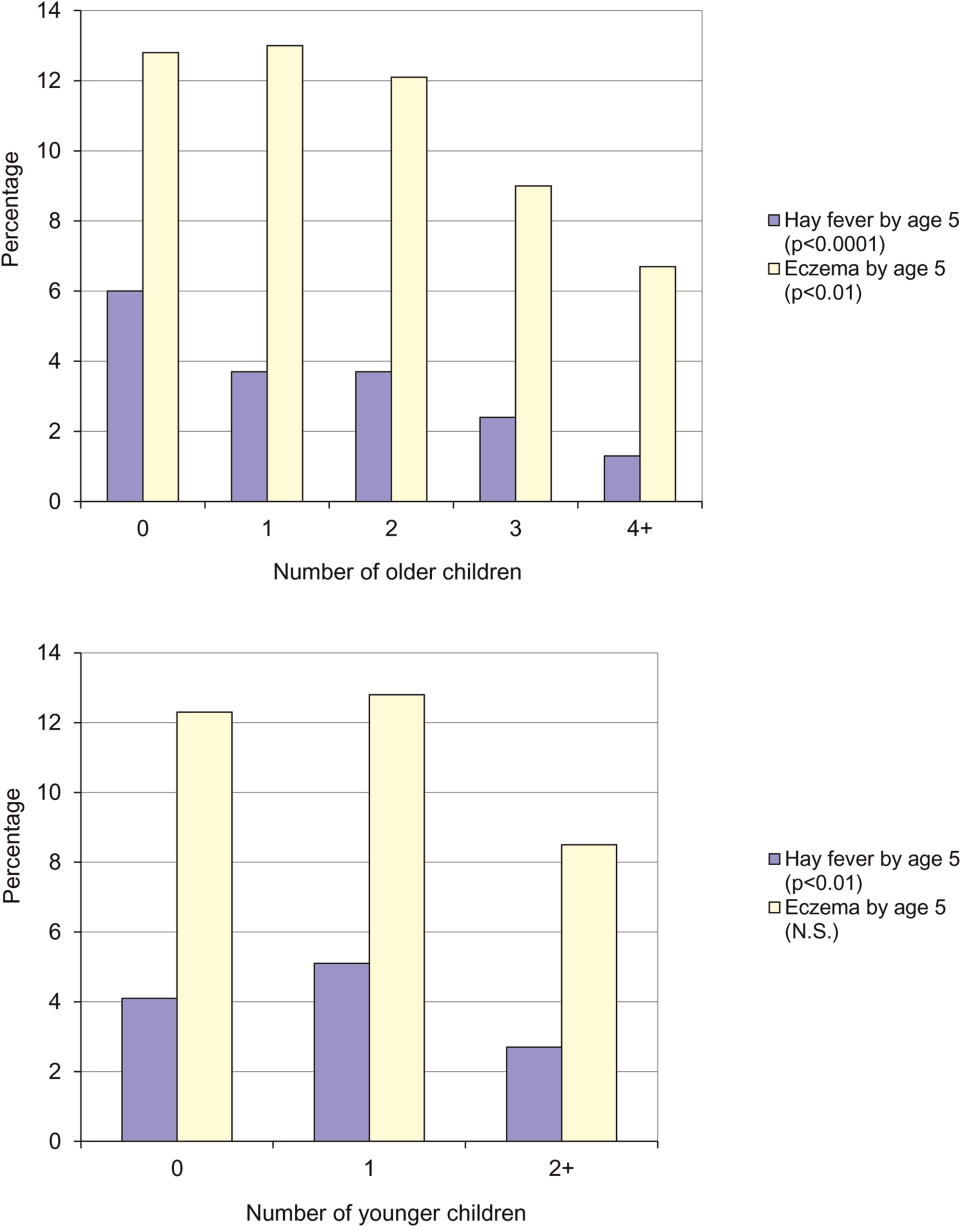
Parent reported eczema and hay fever by five years of age, by number of older children (top panel) and number of younger children (bottom panel) in the household (8).

In 1986, a year after Barker's initial proposal of an association between increased risk of appendicitis and hygiene, Butler & Golding edited a book based on the results of the Child Health and Educational Study – a national longitudinal study of children born during the week 5–11 April 1970 in England, Wales and Scotland (the 1970 British Births Survey) and assessed at five years of age. In one of the chapters, Golding & Peters (as cited in Strachan's paper) observed the same trends that Strachan subsequently reported (8). Parent reported eczema was significantly more common than parent reported hay fever (12.3% vs. 4.4%) and both conditions were inversely related to the number of older (Figure 3, top panel) or younger (Figure 3, bottom panel) children in the household. No association was found between the number of children in the household and the risk of asthma and wheezing.

Golding & Peters concluded that the presence of younger or older siblings appeared to have a similar effect and that it was, perhaps, the total number of children that was most relevant. In the final chapter Golding & Butler state that:

“*either the mother with many children is unlikely to notice that the child is frequently sneezing…or there is a genuine reduction in the incidence of the condition in large families”* (9).

With regard to the trend for eczema not being linear (i.e., the highest prevalence being in those with one sibling), they offered the following explanation:

“*suppose the genuine incidence of a condition rose with the number of children in the family, but that the mother*”*s memory of, or perception of, the condition fell as the number of children increased, one might obtain the sort of pattern shown for eczema…”*.

In summary, at the time of Strachan's paper, the term “hygiene hypothesis” was already in existence (Table 1). However, Golding & Peters had offered no aetiological explanation for the family size effect. In contrast, Barker had postulated that decreased exposure to infections in early childhood had resulted in increased susceptibility to appendicitis because of infections experienced in later childhood. A family size effect was observed, but in the opposite direction to that for hay fever and eczema. No explanation was offered by Barker for the family size effect.

**Table 1:** Comparison of formative papers based on national British birth cohorts for the emergence of the hygiene hypothesis.

	Golding & Peters 1986	Barker 1988	Strachan 1989
Cohort studied	1970 British Births Survey	1946 National Survey of Health and Development 1958 National Child Development Study 1970 British Births Survey	1958 National Child Development Study
Outcome	Hay fever (parent reported by 5 years) Eczema (parent reported by 5 years)	Appendicectomy	Hay fever (self reported in last 12 months at age 23) Hay fever (parent reported in last 12 months at age 11) Eczema (parent recall when child aged 7 of presence in the first year of life)
Family size effect	Decreased hay fever and eczema with increased sibship size	Increased risk of appendicectomy with increased sibship size	Decreased hay fever and eczema with increased sibship size
Older versus younger siblings	Effect considered to be similar	Not differentiated	Effect stronger for older siblings
Aetiological explanation for sibship effect	Not postulated	Not postulated. Increased sibship size regarded as a marker of overcrowding.	Sibship effect could be explained if allergic diseases were prevented by infection in early childhood transmitted by unhygienic contact with older siblings, or acquired prenatally from a mother infected by contact with her older children. Later infection or reinfection by younger siblings might confer additional protection against hay fever.
Hygiene hypothesis	Term not used	"Hygiene hypothesis” term used and proposed as an explanation for the rise in appendicitis in Britain during the first half of this century and the continuous fall thereafter. As hygiene improved young children began to escape infection and thereby became more vulnerable to appendicitis when exposed to infections in later childhood and early adult life. The hygiene hypothesis, it was suggested, would mean that with continued improvements in hygiene, exposure to infections during childhood and early adult life falls and rates of appendicitis fall with it.	“Unhygienic contact with older siblings” stated, but term “hygiene hypothesis” not used in the paper
Type of infection involved	Infections not mentioned	Postulated that “both respiratory and enteric infections cause appendicitis”	Type of infections not stipulated

**Figure 4:**
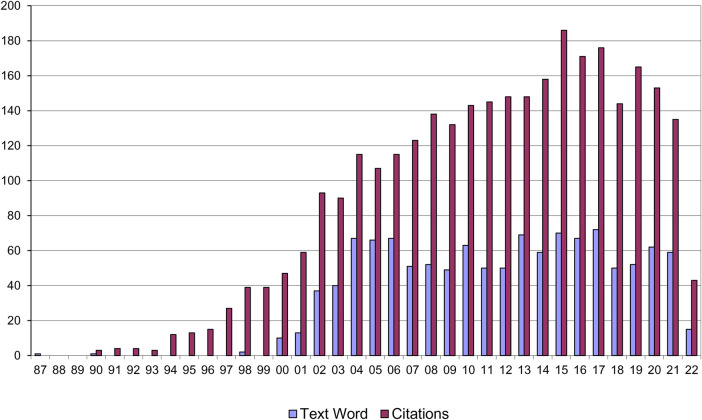
Citations to Strachan's *“*hygiene hypothesis” paper and papers using the term *“*hygiene hypothesis” in the title or abstract.

Since its publication Strachan's paper has become a citation classic (3,093 citations as of 17/05/22) (Figure 4) reflecting an exponential interest in the hypothesis and its extension into a diversity of areas of research.

## Hygiene hypothesis – emergence of an immunological basis

An immunological basis for the hygiene hypothesis subsequently emerged (10, 11). Romagnani proposed that atopy was a T-helper 2 (Th2) cell driven hypersensitivity to innocuous antigens (allergens) (12). Subsets of T-helper (Th) cells produce different patterns of cytokines (13). Th1 cells play a critical role in protection against viral, bacterial and fungal pathogens. Th2 cells are responsible for protection from helminthic infections (14). Th2 cells control IgE production *via* IL-4 and interleukin-13 (IL-13) production (14). The foetus in containing paternally derived cells is essentially an allograft. In order to survive it is essential to suppress Th1 immunity. This suppression provides a strong anti-cytotoxic stimulus preventing lysis of incompatible foetal tissue (14). Thus, during pregnancy, up regulation of Th2 T-cell function appears to be an important prerequisite for successful pregnancy (14). Following birth, genetic and environmental factors interact to determine whether an infant continues with a Th2 predominant pattern of cytokine responses, as is found in atopic individuals; or whether the child develops a Th1 pattern associated with non-atopy (15–17). Microbiological factors appear to be crucial in determining whether this Th2 to Th1 switch occurs. Animal models have shown that a microbe-free environment locks the immune system into a Th2 state (18).

The degree to which the Th1/Th2 paradigm alone can account for the hygiene hypothesis effect has waned over time. In 2004 Sheikh and Strachan concluded:

“The earlier suggestion of a reciprocal relation between Th1- and Th2-mediated conditions now, however, seems somewhat simplistic, not least because autoimmune and atopic allergic disorders frequently coexist within the same individual. More plausible, perhaps, is the recent suggestion that reduced exposure to infectious agents during early childhood might exert a profound adverse effect on the developing immune system through effects on regulatory T cells, leading to the generation of inappropriate immunologic responses to autoantigens (manifesting as autoimmune disease), allergens (manifesting as atopic allergy), or both” (19).

## Hygiene hypothesis and objective measures of atopy

The early reports by Strachan (1) and Golding & Peters (8) were based on self-report and/or parental report. The possibility that a mother's memory of, or perception of the atopic conditions might vary with the number of children she has precipitated the interest in determining whether the sibling size effect existed for objective measures of atopy. The World Allergy Organization define atopy as:

“a personal and/or familial tendency, usually in childhood or adolescence, to become sensitised and produce IgE antibodies in response to ordinary exposures to allergens, usually proteins. As a consequence, these persons can develop typical symptoms of asthma, rhinoconjunctivitis, or eczema” (20).

The WAO state that IgE sensitisation can be documented by IgE antibodies in serum or by a positive skin prick test (20). A series of studies subsequently emerged confirming inverse correlations between sibship size and objective measures of allergic sensitisation, either skin prick testing (21–24) or specific IgE (25–27), arguing strongly against a spurious relationship attributable to differential recognition, reporting or diagnosis of allergic symptoms in smaller and larger families (28).

## Hygiene hypothesis and affluence

Within a developed country such as the UK the sibship size effect is observed across social class groups. Using data from the National Child Development Study, the prevalence of hay fever declined with increasing family size and this effect was apparent across social class groups. Independently hay fever was lower among the offspring of unskilled and semi-skilled manual workers (IV & V) than those of skilled workers (III) or professional, managerial and intermediate groups (I & II) (29).

## Hygiene hypothesis around the world – developed vs. developing world

The initial hygiene hypothesis paper was based on a birth cohort study from the UK. Whilst the sibling size protective effect was subsequently replicated in a number of developed countries there was a paucity of data to establish whether the effect was observed in less affluent countries. More recently Strachan et al. used questionnaire data from Phase Three of the International Study of Asthma and Allergies in Childhood (ISAAC) including 210 200 children aged 6–7 years from 31 countries, and 337 226 children aged 13–14 years from 52 countries (30). Associations of disease symptoms and labels of asthma, rhinoconjunctivitis and eczema were analysed by numbers of total, older and younger siblings, using mixed (multi-level) logistic regression models to adjust for individual covariates and at the centre level for region, language and national affluence. In both age groups, inverse trends were observed for reported “hay fever ever” and “eczema ever” with increasing numbers of total siblings, and more specifically older siblings. These inverse associations were significantly stronger in more affluent countries. In contrast, symptoms of severe asthma and severe eczema were positively associated with total sibship size in both age groups. These associations with disease severity were largely independent of position within the sibship and national Gross National Income per capita, suggesting at least two distinct trends: inverse associations with older siblings (observations which prompted the “hygiene hypothesis” for allergic disease) are mainly a phenomenon of more affluent countries, whereas greater severity of symptoms in larger families is globally more widespread.

## Hygiene hypothesis and “pollution”

The sibship size effect being less strong in less affluent countries could potentially reflect the confounding effect of pollution exposure in poorer countries. von Mutius compared the rates of skin prick positivity in 9–11 year old children in Leipzig and Halle in East Germany, and Munich in West Germany. von Mutius noted that the two East German cities were heavily polluted due to private coal burning and industrial emissions. Atopic sensitisation was found to be about three times as prevalent in West Germany as in East Germany. The sibship size effect was present, independent of study area. The contribution of the increased pollution to the lower rate of atopic sensitisation in the East German two cities was not considered in the study (21).

Pollution was considered in a comparison of 10–12 year old children living in urban Konin in Central Poland and rural parts of Sundsvall in northern Sweden. The levels of common industrial pollutants, SO2 and smoke particles were much higher in Konin than in urban Sundsvall and the levels of NO2 were similar. Respiratory symptoms were much more common in the urban Polish children, but these children had a much lower prevalence of sensitisation to allergens, compared with the Swedish children (31).

## Hygiene hypothesis and prevention by “infection”

Nurseries have long been recognised as an environment with a high risk of cross infection. Finnish investigators in 1984 undertook a study of the association between day care attendance and atopy, anticipating that they would find that early cross infection through nursery exposure would increase the risk of allergic symptoms. Finding no difference, they concluded that “atopy probably cannot be prevented by protecting small children from infection.” (32) A subsequent small German study found a reduced risk of allergy among children who had entered day nursery at an early age (33).

At the time of Strachan's 1989 publication, viral infections, particularly of the respiratory tract, were still seen as being potentially important in the development of allergic disease and asthma, as considered in a review paper by Busse (34), cited in Strachan's paper. Larger families with larger number of children inevitably incur more viral infections amongst the children. Hence Strachan's observation of less hay fever in larger families contradicted this previous premise of the role of viral infections in the development of atopic disease. Importantly, the hypothesis Strachan postulated was that “infections” were preventing allergic disease, not specifically or solely viral infections and hence purposely broadening the scope of what might constitute a relevant microbial challenge.

## Hygiene hypothesis - a specific microbial agent or patterns of exposure to infection

Exploring this has come to be one of the significant areas of development of the hypothesis. Subsequent research yielded a number of potential factors that may operate *via* an infectious pathway, however several of these associations have only been found in one study. Infections that may induce a “protective” Th1 cytokine response include: mycobacteria (35), measles (36, 37), hepatitis A (38), Helicobacter pylori (39, 40) and toxoplasma (40).

A potential lack of “protective” infections may be associated with having few siblings (1), excessive “hygiene” (41), antibiotic use in the first two years of life (42), and vaccination of some diseases (42). Not only the infection itself may be important in preventing allergic sensitisation, but also its timing in relation to the first exposure to the relevant allergen (43).

In his review of the hygiene hypothesis, ten years after the initial publication, Strachan noted that attempts to identify specific infections that had inverse associations with sibship size had been largely inconsistent or inconclusive and that:

“*the totality of current evidence from cross sectional and longitudinal studies of common specific and non-specific infectious illnesses in infancy and childhood offers no support for the ‘hygiene hypothesis.’”* (44)

He also noted the move towards studies investigating the influence of the intestinal bowl flora on immunological maturation, citing the pioneering studies comparing infants in Estonia and Sweden and showing marked differences in their commensal gut bacteria, broadly matching the differences seen between atopic and non-atopic infants in each country (45). A year later Matricardi was postulating the significance of high microbial turnover in preventing atopy development (46).

From the turn of the millennium, research interest began to focus on two areas of research which putatively could be argued to have more “unhygienic” environments: the first being the anthroposophic community and the second being the emergence of the farming environment in childhood as being potentially significant.

## Anthroposophic communities

Anthroposophy, also called “spiritual science”, is a spiritual philosophy based on the teachings of Rudolf Steiner, which states that anyone who “conscientiously cultivates sense-free thinking” can attain experience of and insights into the spiritual world. The word anthroposophy is derived from the Greek roots *anthropo* meaning human, and *sophia* meaning wisdom. It has been applied as a philosophy to education (Steiner schools), healthcare, art, architecture, and agriculture (biodynamic farming). Anthroposophical doctors restrict the use of vaccinations, anti-pyretics and antibiotics. The diet often includes the consumption of vegetables preserved by spontaneous fermentation which have been shown to be high in *Lactobacillus plantarum*. Alm assessed the prevalence rates of allergy in two Steiner schools and two local control schools in Sweden (47). The hypothesis was that children who were having fewer vaccinations and therefore more childhood illnesses (e.g., measles) and a diet rich in lactobacilli might have an altered prevalence of atopy. This proved to be the case with an inverse relationship between atopy levels and the number of anthroposophic lifestyle features in the children. No mention is made about unpasteurised milk consumption in either group of children. This study raised significant interest in these children and one of the paper's authors, Scheynius, was a member of the Prevention of Allergy Risk factors for Sensitization In children related to Farming and Anthroposophic Lifestyle (PARSIFAL) research group (48). The PARSIFAL study represented the largest multi-national study of both farming and Steiner children and investigated which factors may contribute to a lower risk of allergy amongst these children (49). Specific factors that were found to be associated with allergic disease included the use of antibiotics in the first year of life (which was associated with an increased risk of every allergic disease but not atopic sensitization) and the use of antipyretics (increased risk of doctor's diagnosis of asthma and current eczema symptoms). Measles infection was not associated with any single allergic outcome on its own, but was associated with less life time eczema and current eczema symptoms combined with IgE sensitization. MMR vaccination was associated with less current and life time rhinoconjunctivitis.

## Farming environment

Man is estimated to have been farming in the United Kingdom for at least 6,500 years. For all but the last two centuries the great majority of the population were involved in agriculture, working directly on the land in close proximity to their crops and animals. However the industrial revolution triggered a mass migration into towns and cities. The perception of the health of farmers has also altered with time. In the latter part of the twentieth century farms have been perceived as being generally “dirty” environments. This “dirt” has historically been seen principally as a hazard, such that in 1998 the American Thoracic Society (ATS) published a 76 page supplement (with 972 references) in the American Journal of Respiratory and Critical Care Medicine, entitled “Respiratory Health Hazards in Agriculture” (50). This opens with the statement:

“*Respiratory diseases associated with agriculture were one of the first-recognised occupational hazards. As early as 1555, Olaus Magnus warned about the dangers of inhaling grain dusts, and the risk was again noted in 1700 by Ramazzini in his seminal work De Morbis Artificum.”*

And subsequently states:

“*Because agriculture is so intimately tied with the land, it has generated many myths about the health of farmers. The long-standing ‘agrarian myth’ was exemplified in Thomas Jefferson's declaration that ‘Cultivators of the earth are the most valuable citizens. They are the most vigorous, the most independent, the most virtuous, and they are tied to their country and wedded to its liberty and interests by the most lasting bonds’. Unfortunately, the myth of the robust, reliably healthy farmer was in actuality a myth that does not correspond with the realities of agricultural life.”*

However, Jefferson was not alone in commenting on the perceived health of farmers. He made the declaration, quoted above, in a letter to John Jay in 1785. A century later, Charles Blackley, the physician who first identified grass pollen as the trigger for hay fever, was commenting:

“It would seem that hay-fever has, of late years, been considerably on the increase….The persons who are most subjected to the action of pollen belong to the class that furnishes the fewest cases of the disorder, namely, the farming class” (51).

## Farmers' children

A number of studies of allergy prevalence in farming children have been undertaken around the world, commencing with a number focussing on the Alpine countries where, traditionally, farming has been the main source of subsistence. Consistently children from rural areas who grow up on farms are at a significantly lower risk of developing atopic conditions than children who live in the same rural area but do not grow up on farms. Farms are rich microbial environments and a stronger protective effect has been observed for children growing up on livestock farms than arable farms.

Two systematic reviews have been published (52, 53). Genuniet identified 39 studies and meta-analysis showed a 25% reduction in asthma prevalence among exposed subjects compared with unexposed subjects (52). Campbell specifically investigated the protective effect of the farming environment on objective markers of atopy (skin prick or specific IgE sensitisation) with 29 farming studies being identified for review (53). A meta-analysis showed a significant protective effect of farm exposure before 1 year of life on allergic sensitization. The specific factors mediating the protective effect appear to be diverse and include endotoxin with farms being environments where high levels of exposure takes place (54), as well as unpasteurised milk consumption with the latter being associated with significantly less current eczema symptoms and a greater reduction in atopy (55). Specifically growing up on a farm does not seem to be required to achieve a protective effect, rural children, both farming and non-farming, have both been found to have reduced atopy through consumption of unpasteurised milk and visits to stables (56).

### Family size and farm exposure

A logical extension from the observation of a protective effect of the farm environment on the development of hay fever and atopy amongst children was to explore the independence and interaction of this protective effect with the protective effect of sibship size. This was done in the GABRIELA study in which questionnaire surveys on farming, asthma, and allergies were conducted in four central European areas among 79,888 6–12-yr-old children (57). Aeroallergen-specific serum IgE was measured in a stratified sample of 8,023 children. Multiple logistic regression was used to compare gradients in allergy prevalence by sibship size across three categories of exposure to farming environments.

The prevalence of hay fever ranged from 2% among farmers' children with more than two siblings to 12% among children with no farm exposure and no siblings. Farming families were larger on average. More siblings and exposure to farming environments independently conferred protection from hay fever and objectively measured atopy. There was no substantial effect modification between family size and exposure to farming environments. The odds ratios for hay fever per additional sibling were 0.79 among unexposed non-farm children, 0.77 among farm-exposed non-farm children, and 0.72 among children from farming families (2df interaction test: *p* = 0.41). The consistency of sibling effects across different levels of farming exposure suggested that different biological mechanisms may underlie these two protective factors. Combinations of a large family and exposure to farming environments markedly reduce the prevalence of hay fever and indicate the strength of its environmental determinants (57).

## Pet ownership

The principal bacterial signalling molecule is endotoxin (lipopolysaccharide - LPS) which causes a strong enhancement of Th1 like responses. Homes with pets have been found to contain higher endotoxin levels (58). An early Swedish study found a reduced prevalence of allergic rhinitis and asthma among children exposed to pets in the first year of life which persisted, albeit at reduced levels of statistical significance, after adjustment for pet avoidance (59). In contrast, a pooled analysis of 11 prospective European birth cohorts totalling 22,000 children found no association between furry and feather pet ownership in early life and asthma or allergic rhinitis at school age (60). Conversely, a systematic review published the same year found that six out of nine included articles found a reduction in allergic disease associated with perinatal exposure to dots, or to cats or dogs (61). Of those studies observing a protective effect, consistently dog exposure appears to confer a greater protective effect on allergy development than cat exposure in early life (27).

## Hygiene hypothesis and microbial exposure as opposed to infection

The Old Friends (OF) Mechanism was proposed by Rook in 2003 and argues that the vital microbial exposures are not colds, measles and other childhood infections (the crowd infections), but rather microbes already present during primate evolution and in hunter-gatherer times when the human immune system was evolving. OF microbes include environmental species which inhabit indoor and outdoor environments, and the largely non-harmful commensal microbes acquired from the skin, gut and respiratory tract of other humans. In evolving humans, before the advent of modern medicine, the OF also included organisms such as helminths, Helicobacter pylori, and hepatitis A virus that could persist for life in hunter gatherer groups and that needed to be tolerated. They all therefore activated immunoregulatory mechanisms, but few experts believe that they need to be replaced or even that there is any feasible way of doing so (62).

## Hygiene hypothesis and gut microbiota

In one of the earliest explorations of the gut microbiota and its association with the development of atopic disease, specifically eczema, in the AllergyFlora study infants recruited in Goteborg, London and Rome had their gut microbiota assessed at multiple time points in the first year of life: 3, 7, 14, and 28 days and 2, 6, and 12 months of age (63). At 18 months of age, atopic eczema and total and food-specific IgE levels were assessed. These outcomes were modelled in relation to time to colonization with 11 bacterial groups and to ratios of strict anaerobic to facultative anaerobic bacteria and gram-positive to gram-negative bacteria at certain time points. Neither atopic eczema nor food-specific IgE by 18 months of age were associated with time of acquisition of any particular bacterial group. Caesarean section delayed colonization by Escherichia coli and Bacteroides and Bifidobacterium species, giving way to, for example, Clostridium species. Lack of older siblings was associated with earlier colonization by Clostridium species and lower strict anaerobic/ facultative anaerobic ratio at 12 months (63).

A nested case control analysis was subsequently undertaken in this same cohort using a culture-independent approach to relate the colonization pattern to development of atopic eczema in the first 18 months of life (64). Faecal samples were collected from 35 infants at 1 week of age. Twenty infants were healthy, and 15 infants were given diagnoses of atopic eczema at the age of 18 months. The faecal microbiota of the infants was compared by means of terminal restriction fragment length polymorphism (T-RFLP) and temporal temperature gradient gel electrophoresis (TTGE) analysis of amplified 16S rRNA genes. By means of T-RFLP analysis, the median number of peaks, Shannon-Wiener index, and Simpson index of diversity were significantly less for infants with atopic eczema than for infants remaining healthy. The same was found when TTGE patterns were compared. The authors concluded that there is a reduced diversity in the early faecal microbiota of infants with atopic eczema during the first 18 months of life (64).

## Helminths

Another manifestation of hygiene levels in a society is helminth carriage. Improved hygiene in developed countries has resulted in diminished endemic carriage of helminths, hence the interest as to whether helminth carriage might be related to protection from developing allergies.

Cooper et al. studied the relation between geohelminth parasites and expression of atopy in a study of school-age children from rural Ecuador (65). They found that active infection with any geohelminth and/or a history of infection with Ancylostoma lumbricoides and Ancylostoma duodenale were associated with significant protective effects against allergen skin test reactivity. Children with the highest levels of total IgE or with anti-A. lumbricoides IgG4 antibodies were also protected against skin test reactivity, and the protective effects of high IgE, anti-A. lumbricoides IgG4 and/or active geohelminth infections were statistically independent.

Strachan's postulation of a protective effect being experienced prenatally from a mother infected during pregnancy has been explored in the context of helminth infections. Cooper et al. sought to investigate the effect of maternal geohelminths on the development of eczema, wheeze, and atopy during the first 3 years of life (66). Maternal stool samples were examined for geohelminths by microscopy and geohelminths were observed in 45.9% of mothers. The authors concluded that their data did not support a protective effect of maternal infections with geohelminth parasites during pregnancy against the development of eczema and wheeze in early childhood, although there was evidence in subgroup analyses for a reduction in SPT reactivity to house dust mites and perennial allergens (66). In a follow-up of the same cohort to 5 years of age, geohelminth infections during childhood were not associated with atopy, as measured by skin prick tests (67).

Flohr et al. explored the association between helminth carriage and sanitary conditions amongst 1,601 Vietnamese schoolchildren aged 6 to 18 years. Sensitization to house dust mite was present in 14.4% and to cockroach in 27.6% of children. House dust mite sensitisation was reduced in those with higher hookworm burden and with Ascaris infection and increased in those using flush toilets. In contrast, sensitization to cockroach was not independently related to geohelminth infection but was increased in those regularly drinking piped or well water rather than from a stream (68).

In an elegant RCT in the same cohort, the hypothesis that helminths protects against allergic disease and allergen skin sensitization was tested by randomly allocating 1,566 schoolchildren aged 6–17 to receive either anti-helminthic therapy or a placebo at 0, 3, 6, and 9 months (69). Antihelminth therapy had no effect on exercise-induced bronchoconstriction, questionnaire-reported wheeze or rhinitis, or flexural dermatitis on skin examination. However, anti-helminthic therapy was associated with a significantly higher allergen skin sensitization risk, particularly among children infected with A. lumbricoides at baseline. The authors concluded that a significant reduction in worm burden (any helminth prevalence reduced from 67.4% at baseline to 9.2% after treatment over a 12-month period) in helminth-infected children increases the risk of allergen skin sensitization but not of clinical allergic disease (69).

## Hygiene hypothesis and genetic variants

The substantial increase in the prevalence of AR has only occurred over the last few decades, a period too short for substantial change in the genetic makeup of populations, alterations in environmental and lifestyle factors must also be important in the pathogenesis of disease. However, a study was undertaken to identify genetic variants that modify the protective effect of increasing birth order and AR and IgE sensitization to grass using genome-wide association study data from approximately 13,000 subjects taking part in four large European cohort studies (70). No statistically significant interaction effects were found (70).

## Robustness of the birth order effect finding

The birth order effect on atopy has proved remarkably robust. Despite the large number of citations of the hygiene hypothesis paper, the last published systematic review of the sibship size effect was in 2002 (71), although another is under way (72). In the 2002 review, 53 different studies were identified. For eczema, 9 of 11 studies reported an inverse relation with number of siblings; for asthma and wheezing, 21 of 31 reported the inverse association; for hay fever, all 17 studies showed the effect; for allergic sensitisation or immunoglobulin E reactivity 14 of 16 studies supported the “protective” effect of a higher number of siblings (71).

### Older vs. younger siblings

Generally the association has been found to be stronger for number of older siblings than for number of younger siblings. But the independent inverse association between AR or atopy and number of younger siblings is highly significant because it precludes the birth order effect being attributable exclusively to any kind of adaption of the maternal-foetal interface with a mother's successive pregnancies.

### Birth spacing observation

In one study a trend towards a lower prevalence of doctor diagnosed hay fever was observed with closer birth spacing, but this was of only borderline significance (0 05 < *p* < 0 1) after adjustment for other factors (22).

### Sibling gender observation

In some studies exploring the sibship size effect a significantly stronger protective effect has emerged for brothers than for sisters (27, 73).

### Maternal age observation

Two studies have shown an increasing prevalence of allergic symptoms among the offspring of older mothers, after adjustment for family size and birth order (22, 29).

## Other exposures that have been considered to constitute aspects of the hygiene hypothesis

The list of exposures that are considered to be consistent with some aspect of the hygiene hypothesis are protean. Barker, investigating the aetiology of appendicitis, explored the number of people per room in a household as a proxy for overcrowding and hence increased likelihood of cross infection between family members (7). With regard to atopy, a European study found an additional protective effect of sharing a bedroom as a child, independent of family size (27).

The microbial content of drinking water has also been investigated, comparing schools in the Karelian regions of Finland and Russia. Finnish school children were much more likely to be sensitised to pollens and specifically birth, but total numbers of microbials cells in drinking water samples were substantially higher in the Russian schools compared with the Finnish schools. The authors concluded that the high microbial content of drinking water might be reducing the risk of atopy (74).

Consistent with the observation of a protective effect from enhanced faecal microbial diversity observed in the AllergyFlora study, further research in the Finnish Karelia established that healthy teenagers had higher environmental biodiversity with more species of plants around their homes and higher generic diversity of Gram-negative Gammaproteobacteria on their skin compared with allergic subjects (75).

## Hygiene hypothesis and “cleanliness”

As noted previously, the term “hygiene hypothesis” was already existent at the time of Strachan's 1989 publication, but that paper did not include the term. The word “hygiene” was present in the title of the report “Hay fever, hygiene and household size” and within the text of the report it did state “…..if allergic diseases were prevented by infection in early childhood, transmitted by *unhygienic* contact with older siblings….” and subsequently that “…improvements in household amenities, and higher standards of personal *cleanliness* have reduced the opportunity for cross infection in young families”. Hence, it can be seen how the term hygiene hypothesis was so readily linked to Strachan's explanation for his findings. Furthermore, it is also apparent how the controversy about the role of cleanliness and allergy development subsequently arose.

Few studies have explored aspects of cleanliness and the likelihood of allergies developing. In the Avon Longitudinal study of Parents and Children birth cohort study of 14,541 pregnancies, there is some limited evidence for a relationship between hygiene and eczema. At age 15 months, a “hygiene score” was derived from parent report of the frequency of face washing, hand washing/wiping, hand cleaning pre-meals and bathing or showering (76). Wheezing and atopic eczema at 30 to 42 months of age were both weakly but significantly more common in families with high hygiene scores (77). The authors concluded:

“*The importance of hygiene in public health should not be dismissed; however, the creation of a sterile environment through excessive cleanliness may potentially be harmful to the immune system.”*

An Australian study explored the association between dummy (pacifier) use in infancy and sanitisation of the pacifier. Any pacifier use at 6 months was associated with a doubling of risk of food allergy, mainly attributable to a high risk among infants using pacifiers washed with antiseptic. Food allergy was not increased among those using pacifiers without antiseptic at age 6 months, nor among infants using any form of pacifier at other ages (78).

## Criticisms of the hygiene hypothesis

A concern has been raised that the term “hygiene hypothesis” has encouraged the benign neglect or deliberate avoidance of important hygiene measures for the prevention of infectious disease. In a paper entitled “Time to abandon the hygiene hypothesis….” Bloomfield states:

“*Interaction with microbes that inhabit the natural environment and human microbiome plays an essential role in immune regulation. Changes in lifestyle and environmental exposure, rapid urbanisation, altered diet and antibiotic use have had profound effects on the human microbiome, leading to failure of immunotolerance and increased risk of allergic disease*” (62).

Bloomfield concludes:

“*The term ‘hygiene hypothesis’ must be abandoned. Promotion of a risk assessment approach (targeted hygiene) provides a framework for maximising protection against pathogen exposure while allowing spread of essential microbes between family members*.”

This reiterates the authors earlier desire that the name for the hypothesis move away from “hygiene” to “microbial exposure” or “microbial deprivation” hypothesis. Furthermore, avoiding the term “hygiene” would help focus attention on determining the true impact of microbes on atopic diseases, while minimizing risks of discouraging good hygiene practice (79).

## Family size effect – statistical considerations

In 1997 Strachan wrote:

“*It is almost certain that family size and structure are imprecise surrogate measures for some more influential exposure. If so, the effect of this unknown factor must be substantial. Consider a hypothetical protective agent to which 10% of single child families are exposed, the prevalence of exposure increasing by 10% with each additional child in the family to 50% among families with five children. If exposure to this factor reduced the risk of allergy by a factor of 10 (relative risk 0.1) then the prevalence of allergy would be reduced by about 10% in relative terms for each additional child in the family (relative risk 0.9 per sibling). This is typical of the gradient observed in large epidemiological studies. A similar relationship between sibship size and allergy could be generated by a protective factor with relative risk 0.2 and prevalence ranging in a graded fashion from 30% in single child families to 70% in families with five children*.” (28)

In an elegant analysis, Wickens et al. determined the change in asthma and hay fever that might be expected to be observed based on the decline in family size that had occurred in England & Wales and in New Zealand between 1961 and 1991 (80). The expected relative increase in the prevalence of asthma between 1961 and 1991 as a result of the smaller family size was 1% and 5% for England/Wales and New Zealand, respectively; smaller family size would be expected to increase the prevalence of hay fever prevalence in England/Wales by 4% (80). The authors concluded that:

“*Changes in family size over the last 30 years do not appear to explain much of the reported increase in asthma or hay fever prevalence.”* (80)

## Hygiene hypothesis and other conditions

The underlying premise of the hygiene hypothesis, that a decrease in infectious burden has been associated with an increase in atopy, has been extended to other conditions including autoimmune diseases (81). Increasing duration of contact with a younger sibling aged less than 2 years in the first 6 years of life was associated with reduced multiple sclerosis *n* (82). Similarly, exploring the aetiology of childhood IDDM in Northern Island, the lowest incidence was observed in areas with highest population density and most household crowding with the explanation being offered that exposure to infections very early in childhood is the protective factor and that later infections may act as initiators or promoters of diabetes (83). This exact same explanation has also been offered with regard to the aetiology of childhood acute lymphoblastic leukaemia: Microbial exposures earlier in life have been postulated as being protective but, in their absence, later infections trigger the critical secondary mutations resulting in ALL development (84). The leading idea is that some infectious agents – notably those that co-evolved with us – are able to protect against a large spectrum of immune-related disorders dependent on a child experiencing them early in childhood (81).

## Hygiene hypothesis – is it an *in utero* phenomena?

Strachan raised the suggestion in the original report that *in utero* infections might be responsible for the older sibling effect. However it might not necessarily be *in utero* infections - the maternal-foetal interface changes significantly with additional pregnancies. This is best exemplified in pre-eclampsia, generally thought to be a disease mainly of first pregnancies (85). Karmaus explored the relationship between cord blood IgE levels and birth order. Using ordinal regression, the authors found that IgE is reduced with increasing birth order (first child: OR = 1; second child: OR = 0.78, 95% CI: 0.57–1.05; third child: OR = 0.59, 95% CI: 0.41–0.83) and concluded that the sibling effect may have its origin *in utero* (86).

## The end of the beginning….?

### Serendipity

Sometimes a single paper captures the imagination of the academic community and becomes a seminal moment when a new paradigm is embraced, triggering new areas of research. Golding & Peters had observed the sibship effect before Strachan but had not made any inferences as to its aetiology. Barker had first used the term “hygiene hypothesis” before Strachan, in the context of the aetiology of appendicitis, but his interpretation of the hygiene effect was different. He believed that improved hygiene had led to children experiencing infections later in childhood that were then triggering appendicitis. However, in order to account for the fact that appendicectomy rates had then gone down over time in developed countries, he hypothesised that the ongoing improvements in hygiene were reducing infections in later childhood as well and hence were no longer occurring during the key susceptibility period for appendicitis, the second decade of life. Strachan, in contrast, was the first to postulate that infections were potentially protective for developing atopy.

### Evolution

In science a hypothesis often evolves over time. As the search for specific infections that might be conferring a protective effect proved essentially fruitless, attention began to turn to microbial exposure more broadly, including environments that confer significant microbial exposure (farms, barn and stables), extrinsic vehicles of enhanced microbial exposure (unpasteurised milk consumption, drinking water and pets) and intrinsic exposures (bowel flora). The hygiene hypothesis has led the way with regards to a fundamental reappraisal of our relationship with our microbial environment and that exposure, not avoidance, is what contributes to a child developing a healthy immune system.

### Exposure instead of avoidance

The paradigm of the benefits of exposure has translated into other areas. With regards to aeroallergen exposure a stringent environmental control intervention during pregnancy and early life to create an environment with low levels of house dust mite, cat and dog allergens was found to increase the risk of house dust mite sensitisation at 3 years of age (87). In contrast, the Finnish Allergy Programme (2008–1018) encapsulated this new paradigm with the programme emphasising tolerance rather than avoidance. Primary prevention measures included to not avoid exposure to environmental allergens (foods, pets) and to strengthen immunity by increasing contact with natural environments and encouraging the consumption of probiotic bacteria in fermented foods (88).

With respect to children's diet, in 2000 the American Academy of Pediatrics was recommending for infants at high risk of developing allergies (identified by a strong family history of allergy) that breastfeeding mothers should eliminate peanuts and tree nuts (eg, almonds, walnuts, etc) and consider eliminating eggs, cow's milk, fish, and perhaps other foods from their diets while nursing. Solid foods should not be introduced into the diet of high-risk infants until 6 months of age, with dairy products delayed until 1 year, eggs until 2 years, and peanuts, nuts, and fish until 3 years of age (89). However, two recent seminal studies have demonstrated that infants who have allergenic foods introduced into their diet early are protected from developing food allergy (90, 91).

### Nomenclature

The word “hygiene” has been contentious. Excessive hygiene may contribute to atopy development, but core levels of hygiene are fundamental to personal and community health. Since the Strachan paper alternative terms have emerged. In addition to the “Old Friends Hypothesis” (Rook) (92), these include the “high turnover and diversity hypothesis” (Matricardi) (46), the “microbial deprivation hypothesis” (Björkstén) (93) and the “biodiversity hypothesis” (Haahtela) (94). One of the most interesting areas of research in the coming decade will be to determine the behavioural and microbial impacts of the increased hygiene measures during the Covid pandemic and whether this proves to have any effect on the prevalence of atopy in the cohort of children, and particularly infants, affected by this period.

### Enigma

Despite the shift towards much broader theme of microbial exposure, over 30 years on from the original 1989 publication the sibship size finding remains an enigma. Efforts to identify a specific aetiology through exploring our microbial exposures, evolutionarily old or new, have failed. More intriguing still is the appreciation that the sibship effect now appears to extend into a much broader range of conditions than just atopy. Does this mean that microbes are responsible for these effects too? Or is the older sibship size effect ultimately a reflection of the powerful evolution of maternal foetal interaction with successive pregnancies? A career of investigation on from its original description, the underlying mechanism for variations in allergy prevalence with sibship size and birth order remains, in Churchillian terms, “a riddle wrapped in a mystery inside an enigma”.
